# Plasma Brain-Derived Neurotrophic Factor (BDNF) Concentration and *BDNF*/*TrkB* Gene Polymorphisms in Croatian Adults with Asthma

**DOI:** 10.3390/jpm10040189

**Published:** 2020-10-24

**Authors:** Katherina B. Sreter, Sanja Popovic-Grle, Marina Lampalo, Marcela Konjevod, Lucija Tudor, Matea Nikolac Perkovic, Irena Jukic, Jasna Bingulac-Popovic, Hana Safic Stanic, Jasenka Markeljevic, Nela Pivac, Dubravka Svob Strac

**Affiliations:** 1 Department of Clinical Immunology, Pulmonology and Rheumatology, University Hospital Centre “Sestre Milosrdnice”, 10000 Zagreb, Croatia; ksreter@yahoo.com (K.B.S.); j.markeljevic@gmail.com (J.M.); 2 Clinic for Lung Diseases Jordanovac, University Hospital Centre Zagreb, 10000 Zagreb, Croatia; spopovi1@kbc-zagreb.hr (S.P.-G.); marina.lampalo@gmail.com (M.L.); 3 School of Medicine, University of Zagreb, 10000 Zagreb, Croatia; 4 Division of Molecular Medicine, Rudjer Boskovic Institute, 10000 Zagreb, Croatia; Marcela.Konjevod@irb.hr (M.K.); Lucija.Tudor@irb.hr (L.T.); mnikolac@irb.hr (M.N.P.); npivac@irb.hr (N.P.); 5 Croatian Institute of Transfusion Medicine, 10000 Zagreb, Croatia; irena.jukic@hztm.hr (I.J.); jasna.bingulac-popovic@hztm.hr (J.B.-P.); hsafic@gmail.com (H.S.S.); 6 Faculty of Medicine, Josip Juraj Strossmayer University of Osijek, 31000 Osijek, Croatia

**Keywords:** asthma, plasma BDNF concentration, *BDNF* and *NTRK2* (*TrkB* gene) polymorphisms, severity, phenotypes, aspirin

## Abstract

Brain-derived neurotrophic factor (BDNF) and its tropomyosin-related kinase B (TrkB) receptor might contribute to normal lung functioning and immune responses; however, their role in asthma remains unclear. Plasma BDNF concentrations, as well as *BDNF* and *NTRK2* (*TrkB* gene) polymorphisms, were investigated in 120 asthma patients and 120 healthy individuals using enzyme-linked immunosorbent assay and polymerase chain reaction, respectively. The genotype and allele frequencies of *BDNF* Val66Met (rs6265) and *NTRK2* rs1439050 polymorphisms did not differ between healthy individuals and asthma patients, nor between patients grouped according to severity or different asthma phenotypes. Although plasma BDNF concentrations were higher among healthy subjects carrying the *BDNF* Val66Met GG genotype compared to the A allele carriers, such differences were not detected in asthma patients, suggesting the influences of other factors. Plasma BDNF concentration was not affected by *NTRK2* rs1439050 polymorphism. Asthma patients had higher plasma BDNF concentrations than control subjects; however, no differences were found between patients subdivided according to asthma severity, or Type-2, allergic, and eosinophilic asthma. Higher plasma BDNF levels were observed in asthma patients with aspirin sensitivity and aspirin-exacerbated respiratory disease. These results suggest that plasma BDNF may serve as a potential peripheral biomarker for asthma, particularly asthma with aspirin sensitivity.

## 1. Introduction

Asthma is a chronic inflammatory disease of the airways characterized by the hallmark features of airway hyperresponsiveness, enhanced bronchial smooth muscle cell contractility, and mucus overproduction [1]. It is very common, affecting approximately 235 million children and adults worldwide [2], and causes tremendous morbidity, mortality, and global socioeconomic burden in both Europe [3] and the United States [4]. The remarkable heterogeneity of asthma is underpinned by its wide range of clinical presentations, disease severity, and responses to therapy, as well as the involvement of different pathophysiological mechanisms and biological pathways [5]. The pathogenesis of asthma is clearly multifactorial, governed by the complicated interactions between genetic, environmental, developmental, and immunological risk factors [6]. The overall etiology of this disease, however, is not yet fully understood and remains to be elucidated.

There is growing evidence in the literature suggesting that the link between inflammation and neuronal dysfunction in asthma is mainly provided by neurotrophins (a family of growth factors), notably brain-derived neurotrophic factor (BDNF) [7]. BDNF actions are mediated via binding to its high affinity receptor located within the cell membrane, tropomyosin-related kinase B (TrkB) [8,9,10], causing the activation of the downstream MAPK, PI3K, and PLCγ signaling pathways [11,12,13]. 

It has been shown that the majority of vagal (sensory) neurons innervating the lungs are BDNF-dependent and can affect the functions of motor neurons [14,15,16]. Both BDNF and its receptor TrkB are expressed by diverse cell types in the lungs, such as airway smooth muscle cells, fibroblasts, airway epithelium, neurons, and immune cells [17,18]. BDNF expression and TrkB signaling, as well as their significance in the normal physiological and pathophysiological processes in the lungs continue to be explored [17]. Specifically, various stimuli affecting intra- and extracellular BDNF concentration have been analyzed with a view to understand the relevance of major variations of BDNF levels in human blood and tissue [19]. A number of studies thus far have sought to investigate the role of BDNF in the airways by assessing its concentration locally and in the peripheral circulation, as well as by determining the association between selected polymorphisms in the *BDNF* and *NTRK2* (*TrkB*) genes and the development of asthma [9,20].

Discrepancies between these studies in asthma patients, however, highlight the need for further research in this area [21,22,23,24]. The functional *BDNF* Val66Met (rs6265) variant seems to be particularly important given that the Met allele has been associated with abnormal cellular trafficking and packaging of pro-BDNF, resulting in a decreased production of mature BDNF protein in neurons [25,26]. On the other hand, the *NTRK2* rs1439050 is an intronic polymorphism, and therefore, does not have a specific reported role [27]. Nevertheless, it may still be associated with asthma, albeit not as a vulnerability factor per se (i.e., directly explaining the phenotype), but rather in linkage disequilibrium with a nearby unidentified functional mutation in the *TrkB* gene [27].

Few reports in the literature up to this point have evaluated the possible influence of *BDNF* and *NTRK2* polymorphic variants on serum and plasma BDNF levels in patients with asthma. However, recent studies conducted mainly on the pediatric population across several different countries and ethnicities have produced opposing results [7,15,20,28,29,30,31,32]. These may be due to inconsistencies in the methodological approaches and types of samples used, for instance, blood (plasma, serum, or platelets), sputum, or bronchoalveolar lavage fluid.

To date, BDNF as a potential blood biomarker in asthma has not yet been studied in a Croatian population. Therefore, the aim of our research was to determine the BDNF concentration in the plasma of asthma patients and control subjects, as well as to investigate the potential association of *BDNF* Val66Met and *NTRK2* rs1439050 polymorphisms with asthma, its severity and various phenotypes in an ethnically homogenous group of Caucasian Croatian adults. We hypothesized that plasma BDNF concentration would be significantly altered in asthma patients compared to healthy subjects, while *BDNF* Val66Met and *NTRK2* rs1439050 polymorphisms would be associated with asthma, disease severity, and certain asthma phenotypes.

## 2. Materials and Methods

### 2.1. Participants

This prospective case-control study included a total of 240 subjects of both genders; (120 adult patients with asthma and a control group of 120 healthy adults). All participants enrolled in the study were unrelated Croatian Caucasians. Patients who were previously diagnosed by a pulmonologist as having asthma, and classified according to asthma severity based on the Global Initiative for Asthma (GINA) classification guidelines [33], were recruited consecutively at their regularly scheduled follow-up visits at the Outpatient Department of the Clinic for Lung Diseases Jordanovac, University Hospital Centre Zagreb, in Zagreb, Croatia, from February 2014 to April 2015 and July 2017 to February 2018. The control group consisted of volunteer blood donors, initially screened for good general health and an unremarkable medical history to meet the eligibility criteria for blood donation. Healthy controls were recruited consecutively by the healthcare staff at the Croatian Institute for Transfusion Medicine in Zagreb, Croatia, during their routine blood donation sessions.

Asthma patients satisfied the following inclusion criteria for enrolment into the study: adults (18 years of age and older); no airway disease besides asthma; no acute exacerbation at time of visit; no history of asthma exacerbation or respiratory infection in the previous four weeks; no immune, infectious, or malignant disease; no serious cardiovascular or gastrointestinal disease, or poorly controlled comorbidity (e.g., hypertension, chronic kidney disease, diabetes mellitus, etc.). Healthy control subjects had no history of asthma or other airway diseases, respiratory symptoms, allergic diseases, or recent infections or inflammation. Asthma patients and healthy adults with a documented diagnosis of neurodegenerative or neuropsychiatric disorders, substance abuse, and/or use of anxiolytics, antidepressants, aspirin, or clopidogrel were excluded from the study.

Data pertaining to age, gender, body mass index (BMI), and smoking were collected for both the healthy controls and asthma patients. BMI, an indicator of relative obesity, was calculated using the formula BMI = weight (kg)/height^2^ (m^2^). Subjects with a BMI of 30 and above were considered obese according to the World Health Organization criteria. In addition, a complete medical history, physical examination, and diagnostic work-up were conducted on each asthma patient. Skin prick testing (SPT) was carried out with common airborne allergens (Stallergen allergenic extracts for skin prick testing with control solution, Paris, France), with positive (histamine) and negative (saline) controls. Spirometry with bronchodilation and single-breath diffusing capacity of lung for carbon monoxide (DLCO) tests were performed using the Master-Screen pulmonary function testing analyzer (Version 5.0, Jaeger GmbH, Hoechberg, Germany) according to the manufacturer’s guidelines. Forced expiratory volume in one second (FEV_1_), forced vital capacity (FVC), and peak expiratory flow (PEF) were calculated from the flow-volume loop as the outcome measures using internally derived references. Predicted values for spirometry and pulmonary diffusing capacity are reference values of the European Community for Coal and Steel [34,35]. The fractional exhaled nitric oxide (FeNO) measurement was determined using Denox 88 Module (Eco Medics AG, Duernten, Switzerland). Blood samples were collected in EDTA tubes, and the eosinophil and neutrophil counts were obtained from automated complete blood counts. Total serum immunoglobulin E (IgE) levels were measured using enzyme amplified chemiluminescent immunoassays (Immulite^®^ 2000XPi, Siemens Healthcare Diagnostics, Erlangen, Germany), on Siemens Immulite^®^ 2000XPi automated analyzer, according to the manufacturer’s instructions.

The asthma patients were subdivided into non-severe (mild to moderate) and severe asthma in keeping with the GINA guidelines [33]. Moreover, they were categorized into non-allergic and allergic asthma based on the patient’s clinical history, physical examination, and SPT or extract-based IgE testing [36]. Asthma patients with (≥300 cells/µL) or without (<300 cells/µL) high blood eosinophil count were classified as having eosinophilic or non-eosinophilic asthma, respectively [37]. Subjects with asthma were also divided into Type 2 (T2)-high and T2-low groups according to their phenotypic characteristics [38,39,40]. Patients were identified as T2-high if they were allergic (i.e., positive SPT and/or increased level of total IgE (>120 kU/L) or positive specific IgE), had eosinophilic asthma (i.e., high blood eosinophil count ≥ 300 cells/µL), increased value of FeNO > 25 ppb, or exercise-induced asthma (EIA). Patients were considered T2-low if they did not possess any of the aforementioned disease features, but were smokers or obese with BMI > 30 [41]. Patients with the combination of asthma, recurrent nasal polyps, and sensitivity to aspirin or other non-steroidal anti-inflammatory drugs (NSAIDs) were classified as having aspirin-exacerbated respiratory disease (AERD), also known as Samter’s Triad [42], and compared to asthma patients without AERD.

Written informed consent was obtained from each subject prior to participating in the research. The study protocol was approved by the local Ethics Committees of the University Hospital Centre Zagreb, Croatian Institute for Transfusion Medicine, and University of Zagreb, School of Medicine (Project: Person-centered phenotype and genotype research of patients with asthma, Permission No: 02/21 AG). The research was conducted in accordance with the ethical standards laid down by the Declaration of Helsinki of 1975.

### 2.2. Data and Blood Collection

Demographic and clinical data were recorded for each study participant. Whole blood samples were obtained in the mornings (between 7 and 9 a.m.) after an overnight fast to minimize possible circadian variations. Peripheral venous blood samples (8.5 mL) were drawn into tubes containing 1.5 mL of acid-citrate-dextrose anticoagulant solution, immediately refrigerated at a temperature of 4 °C, and then transported to the Rudjer Boskovic Institute in Zagreb, Croatia, for processing within an hour of arrival.

### 2.3. Determination of Plasma BDNF Concentration

Plasma samples for BDNF analysis were obtained by a series of centrifugations of the whole blood. Plasma BDNF concentrations were determined in duplicate by enzyme-linked immunosorbent assay (ELISA) using a commercial kit (Quantikine^®^ ELISA Human Free BDNF Immunoassay, R&D Systems, Minneapolis, MN, USA), according to the manufacturer’s guidelines. After terminating the antibody-enzyme-substrate reaction, the optical density in each well was determined using an ELISA microplate reader set to 450 nm, with wavelength correction set to 570 nm. The BDNF plasma concentrations of the samples in each assayed plate were calculated based on the standard curve. The intra- and inter-assay coefficients of variations were minimal (less than 10%).

### 2.4. DNA Extraction and Genotyping

Genomic DNA was isolated from peripheral blood leukocytes using a salting-out method [43]. The *BDNF* Val66Met (rs6265) and *NTRK2* rs1439050 gene polymorphisms were genotyped with TaqMan SNP Genotyping Assay (Applied Biosystems^®^, Foster City, CA, USA) using real-time polymerase chain reaction (PCR) conducted on an ABI Prism^®^ 7000 Sequence Detection System apparatus according to the manufacturer’s instructions. Briefly, 20 ng of genomic DNA was amplified in a 10-µL reaction volume under the following PCR reaction conditions: 40 cycles at 92 °C for 15 s and 60 °C for 60 s.

### 2.5. Statistical Analysis

Statistical analyses were performed using GraphPad Prism version 4.00 for Windows (GraphPad Software, Inc., San Diego, CA, USA). The data were expressed as number (n) and percentage (%) for categorical data or as median with 25th (Q1) and 75th (Q3) percentiles for numerical (continuous) data. All examined parameters failed the assumption of normality of distribution according to the D’Agostino & Pearson omnibus test. Therefore, non-parametric analyses were used to compare the independent cohorts. Continuous variables were compared using Mann-Whitney U test (for comparison of two groups) or Kruskal-Wallis test with post-hoc Dunn’s multiple comparisons test (for comparison of three or more groups). Correlations were assessed with non-parametric Spearman correlation. Possible deviations from the Hardy-Weinberg equilibrium (HWE) were tested using the goodness of fit χ^2^-test. Genotype and allele frequencies were evaluated by a χ^2^-test of independence or Fisher exact test, respectively.

The obtained results were corrected for multiple testing (five comparisons of asthma phenotypes: non-severe vs. severe; T2-high vs. T2-low; non-allergic vs. allergic; non-eosinophilic vs. eosinophilic; non-AERD vs. AERD) using Bonferroni correction, and statistical significance was defined as *p*-value less than 0.01. A priori sample size and achieved power calculations were conducted using the G*Power 3 Software Version 3.1.9.2. (Free program written by Franz Faul, University of Kiel, Kiel, Germany). For Mann-Whitney test, at medium effect size 0.425, power 0.8, and statistical significance set at 0.01, an adequate total sample size was determined to be 236. For Kruskal-Wallis test (three groups) at medium effect size 0.25, power 0.8, and statistical significance set at 0.01, an adequate sample size was determined to be 228. For χ^2^-test (df = 2), at small-to-medium effect size 0.25, power 0.8, and statistical significance set at 0.01, an adequate sample size was determined to be 223, whereas for Fisher exact test (df = 1) at small-to-medium effect size 0.25, power 0.8, and statistical significance set at 0.01, an adequate sample size was determined to be 187. Given that the actual total sample size was 240, the power analysis confirmed the appropriate sample size and statistical power of the study.

## 3. Results

A total of 120 asthma cases (41 males and 79 females) and 120 control subjects (73 males and 47 females) were examined (Table 1). There were more males (60.83%) in the control group (*p* < 0.0001), while females predominated (*p* < 0.0001) in the asthma group. Asthma patients were significantly older (*p* < 0.0001) than healthy individuals, and this age disparity was due to the significantly older individuals (61.00 years, 53.00–67.50) in the group of severe asthma patients.

**Figure 1:**
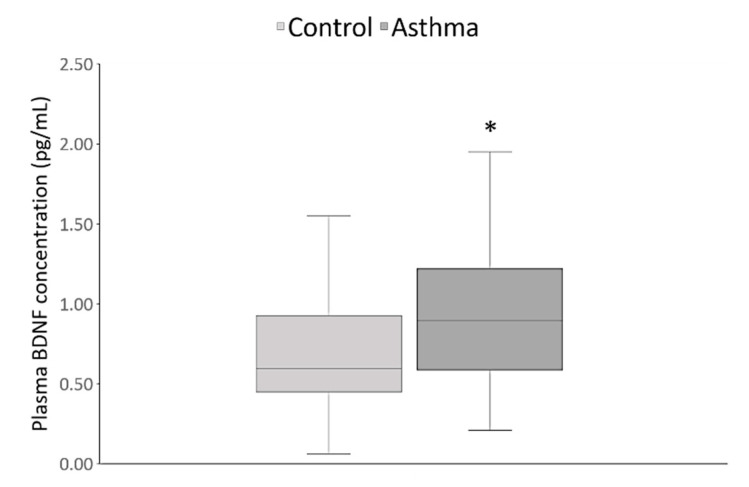
Plasma brain-derived neurotrophic factor (BDNF) concentrations were significantly higher in the asthma patients (0.89 pg/mL, 0.59–1.22) as compared to the healthy subjects (0.59 pg/mL, 0.45–0.92). * *p* < 0.0001, U = 4902.00, Mann-Whitney test.

Specifically, further analysis by Kruskal-Wallis test, followed by Dunn’s multiple comparisons test, demonstrated that the group of severe asthma patients (n = 61) was significantly older than the group (n = 59) of non-severe asthma patients (47.00 years, 35.00–67.00; *p* = 0.005), and the control group (42.00 years, 33.25–51.00; *p* < 0.0001). Significantly fewer patients with asthma (8.33%) than healthy subjects (34.17%) were current smokers (*p* = 0.0005), although non-smokers (never and past smokers) were more prevalent (*p* < 0.0001) in both groups (Table 1). On the other hand, there were no differences in BMI (*p* = 0.64) between asthma cases and healthy controls, even across six different BMI categories (*p* = 0.48), including underweight, normal weight, overweight, and three categories of obesity, or when the subjects were divided into obese or non-obese individuals (*p* = 0.43). As shown in Figure 1, plasma BDNF concentrations were significantly higher (*p* < 0.0001) in asthma patients in comparison to the healthy subjects.

There was no significant correlation between plasma BDNF levels and age in asthma patients (*p* = 0.49, r = 0.06) and control group (*p* = 0.84, r = −0.02). The concentration of BDNF in plasma was not significantly correlated with the BMI of asthma patients (*p* = 0.17, r = 0.13) or healthy subjects (*p* = 0.54, r = 0.06). There were also no significant differences in plasma BDNF levels between smokers and non-smokers in the control (*p* = 0.23) and asthma (*p* = 0.07) groups. When the asthma patients and control subjects were subdivided according to gender, we observed no significant differences in plasma BDNF levels between male and female asthma patients (*p* = 0.67), whereas healthy males had nominally higher BDNF concentrations in plasma than healthy females (*p* = 0.04); however, this difference was not significant after applying the correction for multiple testing. 

The *BDNF* Val66Met genotype distributions were in HWE for both the control group (*p* = 0.38) and asthma patients (*p* = 0.26). As demonstrated in Table 2, the *BDNF* Val66Met genotype (*p* = 0.54) and allele (*p* = 0.35) frequencies did not differ significantly between healthy subjects and asthma patients. A comparison of the carriers of GG homogenous genotype and A allele carriers revealed no significant difference in their distribution (*p* = 0.34) between healthy individuals and asthma patients. Likewise, the G versus AA carrier frequencies did not differ significantly (*p* = 1.00) between these groups (Table 2).

A difference in the plasma BDNF concentrations of the control subjects was observed when the asthma patients and healthy individuals were subdivided according to *BDNF* Val66Met genotype; healthy carriers of the AG genotype were found to have nominally lower BDNF concentrations (*p* = 0.03) than healthy carriers of the GG genotype (Table 3). Moreover, plasma BDNF concentrations in the control group were significantly decreased (*p* = 0.008) among carriers of the A allele when compared to GG carriers. Healthy carriers of the G allele, however, did not have significantly different plasma BDNF concentrations (*p* = 0.65) than the AA genotype carriers.

On the other hand, there were no significant differences in plasma BDNF concentrations (*p* = 0.52) of asthma patients split into groups based on *BDNF* Val66Met genotype (Table 3). There were no significant differences in the plasma BDNF concentrations between asthma patients carrying the A allele and GG genotype (*p* = 0.39), as well as between asthmatic carriers of G allele and AA homozygous genotype (*p* = 0.37). 

The *NTRK2* rs1439050 genotype distributions were also in HWE for both the control group (*p* = 0.34) and the individuals with asthma (*p* = 0.40). The *NTRK2* rs1439050 genotype (*p* = 0.86) and allele (*p* = 0.68) frequencies did not differ significantly between healthy participants and asthma patients (Table 4). As demonstrated in Table 4, the G versus TT carrier frequencies did not differ significantly (*p* = 1.00) between these groups. In addition, no significant difference (*p* = 0.70) in the distribution of GG homogenous genotype and T allele carriers was observed between healthy individuals and those with asthma.

We also investigated whether rs1439050 polymorphic variants, located within the *NTRK2* gene encoding the TrkB receptor, influence the plasma level of unbound/active BDNF protein. As demonstrated in Table 5, there were no significant differences in plasma BDNF concentrations of healthy subjects (*p* = 0.42) or asthma patients (*p* = 0.13) split into groups based on *NTRK2* rs1439050 genotype. There were also no significant differences in the plasma BDNF levels between healthy individuals (*p* = 0.52) or asthma patients (*p* = 0.23) carrying the G allele and TT genotype, as well as between healthy (*p* = 0.36) or asthmatic carriers (*p* = 0.21) of T allele and GG homozygous genotype (Table 5).

**Figure 2:**
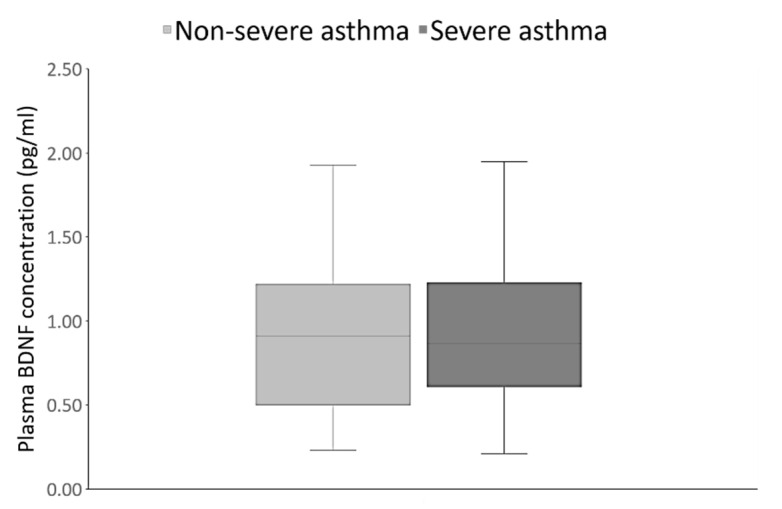
Plasma BDNF concentrations did not differ significantly between the non-severe (0.91 pg/mL, 0.50–1.22) and severe (0.88 pg/mL, 0.61–1.29) asthma patients. *p* = 0.7094, U = 1728.00, Mann Whitney test.

We also examined the relationship between plasma BDNF levels and asthma severity. As shown in Figure 2, plasma BDNF concentrations did not differ significantly between the non-severe and severe asthma patients (*p* = 0.71).

This finding was confirmed by additional analysis with Kruskal-Wallis test, followed by Dunn’s multiple comparisons test. Plasma BDNF levels were significantly higher in both non-severe (0.91 pg/mL, 0.50–1.22; *p* = 0.005) and severe asthma patients (0.88 pg/mL, 0.61–1.29; *p* = 0.0004) compared to the control group (0.56 pg/mL, 0.45–0.92). There were no significant differences in the *BDNF* Val66Met genotype (*p* = 0.13) and allele (*p* = 0.11) frequencies between non-severe and severe asthmatics (Table 6). As shown in Table 6, no significant differences between non-severe and severe asthma patients were observed in the distribution of the carriers of the GG genotype and the A allele (*p* = 0.19), as well as in the frequencies of G allele and AA genotype carriers (*p* = 0.24).

Moreover, there were no significant differences in the *NTRK2* rs1439050 genotype (*p* = 0.56) and allele (*p* = 1.00) frequencies between the non-severe and severe asthma patients (Table 7). As demonstrated in Table 7, no significant differences between the non-severe and severe asthma patients were detected in the distribution of the carriers of the TT genotype and the G allele (*p* = 0.43), as well as in the frequencies of T allele and GG genotype carriers (*p* = 0.72).

Asthma patients demonstrated high heterogeneity in their clinical symptoms and phenotypes as evidenced by the data in their medical records (summarized in Table 8). A positive correlation (*p* = 0.023, r = 0.21) was observed between plasma BDNF concentration and duration of disease in asthma patients (Table 8), although this result was not significant after correcting for multiple testing. On the other hand, asthma patients with a history of pneumonia (0.69 pg/mL, 0.48–0.92) had nominally lower plasma BDNF concentrations (*p* = 0.026) in comparison to asthma patients who had previously never developed pneumonia (0.95 pg/mL, 0.61–1.29). Plasma BDNF concentrations were nominally higher (*p* = 0.025) in asthma patients with nasal polyps (1.12 pg/mL, 0.72–1.35) versus those without (0.81 pg/mL, 0.58–1.15). Furthermore, asthma patients with sensitivity to aspirin (1.22 pg/mL, 0.93–1.77) had significantly higher BDNF levels in plasma (*p* = 0.009) than asthma patients lacking aspirin sensitivity (0.84 pg/mL, 0.57–1.18) (Table 8).

In addition, we investigated the plasma BDNF concentrations in various asthma phenotypes. As shown in Table 9, we found no differences in the plasma BDNF concentrations between asthma patients with T2-high and T2-low (*p* = 0.43), non-allergic and allergic (*p* = 0.41), and eosinophilic versus non-eosinophilic asthma (*p* = 0.54) phenotypes. However, patients with AERD demonstrated nominally higher plasma BDNF concentrations (*p* = 0.017) than those without AERD (Table 9).

There were no significant differences in the distribution of the *BDNF* Val66Met genotypes (*p* = 0.50), alleles (*p* = 0.44), GG vs. A allele carriers (*p* = 0.49), and AA vs. G carriers (*p* = 1.00) between asthma patients with T2-high and T2-low phenotypes. In addition, the frequencies of *BDNF* Val66Met genotypes (*p* = 0.27), alleles (*p* = 0.18), GG vs. A allele carriers (*p* = 0.24), and AA vs. G carriers (*p* = 0.55) were not significantly different between non-allergic and allergic asthma patients. Moreover, we observed no significant differences in the frequencies of *BDNF* Val66Met genotypes (*p* = 0.60), alleles (*p* = 0.62), GG vs. A allele carriers (*p* = 0.71), as well as AA vs. G carriers (*p* = 0.56), between patients with and without eosinophilic asthma. There were also no significant differences in the distribution of the *BDNF* Val66Met genotypes (*p* = 0.86), alleles (*p* = 1.00), GG vs. A allele carriers (*p* = 1.00), as well as AA vs. G carriers (*p* = 1.00), between asthma patients with and without AERD (data available on request).

The distribution of the *NTRK2* rs1439050 genotypes (*p* = 0.14), alleles (*p* = 0.07), GG vs. T allele carriers (*p* = 0.55), and TT vs. G carriers (*p* = 0.34) was not significantly different between asthma patients with T2-high and T2-low phenotypes. Moreover, we observed no significant differences in the frequencies of *NTRK2* rs1439050 genotypes (*p* = 0.09), alleles (*p* = 0.06), GG vs. T allele carriers (*p* = 0.13), and TT vs. G carriers (*p* = 0.09) between non-allergic and allergic asthma patients. The frequencies of *NTRK2* rs1439050 genotypes (*p* = 0.78), alleles (*p* = 0.76), GG vs. T allele carriers (*p* = 0.58), as well as TT vs. G carriers (*p* = 1.00) were not significantly different between patients with and without eosinophilic asthma. In addition, asthma patients with and without AERD did not differ in their distribution of the *NTRK2* rs1439050 genotypes (*p* = 0.77), alleles (*p* = 1.00), GG vs. T allele carriers (*p* = 1.00), as well as TT vs. G carriers (*p* = 1.00) (data available on request).

## 4. Discussion

To the best of our knowledge, the present study is the first to investigate the plasma BDNF, *BDNF* Val66Met and *NTRK2* rs1439050 polymorphisms in adult asthma patients and healthy control subjects of the same ethnic and racial background (i.e., Croatian Caucasians). Our findings, demonstrating that control subjects were significantly younger than asthma patients and consisted mainly of men, are in line with the data about blood donors in Croatia, with a median age of 37–40 years and a male predominance of 83.9% [44,45]. The results of our study, showing that the asthma group was older and consisted predominantly of females, are in keeping with the literature reporting that after puberty, women continue to have a higher burden of asthma than males, and this trend continues well into the 5th decade of life [46,47]. In addition, it is not surprising that the observed age difference is due to the significantly older individuals in the group of severe asthma patients, considering the fact that asthma is more severe in older adults and in patients with longer asthma duration [48]. Our results showing that healthy subjects actively smoked more than asthma patients are not unexpected since many asthma patients (30.83% in our study) quit smoking because of more severe asthma symptoms, in addition to the benefit of smoking cessation in improving lung function [49]. The observed 34.17% of smokers in the control group is consistent with the smoking frequency (37.45%) in the general population of Croatia [50]. On the other hand, the 8.33% of active smokers in the asthma group are in line with the reports of smoking among asthma patients in Brazil and Spain, but much lower than what is seen in many other countries [51].

Regarding BDNF concentration in plasma, our findings are in agreement with the results of our previous study showing no significant effects of age, gender, BMI, or smoking on plasma BDNF values [52]. On the other hand, the study of Pillai et al. (2012) found a negative correlation of plasma BDNF levels with age, as well as lower plasma BDNF levels in females than males, possibly because of the weight differences; however, there was no correlation between BDNF plasma concentration and BMI [53]. In contrast to our data, Lommatzsch et al. (2005) reported a decrease in BDNF levels with increasing age or body weight, and there was a negative correlation of plasma BDNF values with BMI [54]. However, similar to our findings of nominally lower plasma BDNF levels in female versus male healthy subjects, women displayed lower platelet BDNF concentrations in comparison to men in the aforementioned study; although, when matched for weight, there were no significant gender differences regarding BDNF plasma levels [54]. Therefore, it is plausible that the nominally lower plasma BDNF levels seen in our female healthy subjects, showing no significant differences after correction for multiple testing, could be due to the lower weight of women than men in the control group. Regarding the association between smoking and BDNF levels, there are discrepancies in the literature, demonstrating lower [55], higher [56], or unchanged [57] concentrations of BDNF in the serum or plasma of smokers compared to nonsmokers. Though these reports on circulating concentrations of BDNF are inconclusive, it is important to recognize that cigarette smoke, at least partly through oxidative stress, has been shown to increase the expression of BDNF and its receptors in airway smooth muscle cells [58]. This smoke-induced enhancement of neurotrophin release and signaling may also occur in other cell types (e.g., epithelium and immune cells), thus contributing to variations in both circulating and local concentrations [58]. Given that BDNF itself mediates both short-term and long-term effects on airway smooth muscle cells after cigarette smoke exposure [58], we surmise that the BDNF levels of the former smokers in the non-smoking cohorts of our study may have off-set any potential differences in the BDNF concentrations of the never-smokers in both the asthma and control groups.

Our finding that asthma patients had significantly higher concentrations of plasma BDNF than healthy individuals is in contrast to earlier studies that reported no differences in the serum and sputum BDNF levels between healthy subjects and patients with asthma [23,24]. Nevertheless, our data are in line with the report on increased BDNF levels in the serum, platelets, and plasma of asthma patients compared to the control group [21,59]. It has also been shown that children with moderate and severe asthma had higher plasma BDNF levels than age-matched control subjects [22]. Moreover, the study of Freeman et al. (2017) demonstrated higher BDNF expression and secretion in airway smooth muscle of asthma patients compared to patients without asthma [60].

A recent study, showing higher amounts of mature BDNF in the sputum of patients with severe asthma in comparison to healthy subjects, has suggested a possible association of BDNF expression with asthma severity [61]. However, our investigation found that while both the non-severe and severe asthma patients had significantly higher plasma BDNF concentrations than the control subjects, there was no difference in the plasma BDNF levels between these groups of asthma patients. Therefore, our findings imply that BDNF could serve as a potential biomarker of asthma, but not asthma severity. This is in contrast to the previously reported data suggesting BDNF as a potential biomarker for clinical severity of asthma in children [22]. These discrepancies might be explained by the fact that Muller et al. (2010) compared the plasma BDNF of pediatric patients categorized into three groups based on asthma severity (mild, moderate, and severe) [22], whereas our study examined the plasma BDNF levels of non-severe (mild and moderate) versus severe asthma in adult patients. 

On the other hand, the findings of our study are in line with the data of Szczepankiewicz et al. (2012), demonstrating similar serum BDNF levels in children with asthma of different severity, regardless of symptom activity [62]. Although differences in plasma BDNF concentration between non-severe and severe asthma patients were not observed in our study, there was a nominally positive correlation between plasma BDNF concentration and duration of asthma. The latter is usually also related to the severity of the disease. However, some studies reported no correlation of serum BDNF levels with asthma duration [63].

In our study, asthma patients with a history of pneumonia had nominally lower plasma BDNF concentrations in comparison to asthma patients who had never developed pneumonia. Lommatsch and co-authors (2007), however, reported markedly reduced BDNF concentrations in the serum and platelets, but not plasma, of patients with acute bacterial lower respiratory tract infection, with normalization to control BDNF levels after antibiotic treatment [64]. Previous studies regarding the effect of viral infections on peripheral BDNF levels were contradictory, with the most recent research by Azoulay et al. (2020) revealing lower serum BDNF levels in patients with severe or moderate SARS-CoV-2 infection, followed by restoration of BDNF levels as patients recovered [65]. Further research is required to clarify the acute and long-term effects of respiratory tract infections on peripheral BDNF levels in the general population, and specifically, in asthma patients. 

Interestingly, we observed the opposite trend of increased plasma BDNF levels in asthma patients with nasal polyps, as well as in those with aspirin sensitivity. As pertains to nasal polyps, Coffey et al. (2009) contrarily reported a decreased mean BDNF concentration, albeit in the sinus mucosa of patients with chronic rhinosinusitis compared to controls, and patients with nasal polyps had the most significant decrease [66]. On the other hand, our results are consistent with the findings of Jornot and colleagues (2007) in nasal polyps, showing that polyp epithelial cells expressed higher BDNF levels compared to turbinate-derived cells, and that BDNF secretion and production were markedly increased in response to pro-inflammatory cytokines [67]. As far as we are aware, there are currently no published data on BDNF levels in relation to aspirin sensitivity. More research is needed to define the role of BDNF in nasal polyps and aspirin sensitivity in asthma. 

When patients were subdivided into those with or without AERD according to the presence or absence of the triad of clinical features (asthma, recurrent nasal polyps, and sensitivity to aspirin or other NSAIDs), nominally higher BDNF levels were observed in patients with AERD. To the best of our knowledge, there are no published data on BDNF levels in AERD patients. The importance of this asthma phenotype is underscored by the increased associated morbidity and costs, as well as its prevalence of 7.15% in asthma patients, as confirmed in our study (7.5%), and 14.89% in patients with severe asthma [68].

Plasma BDNF concentrations in our study did not differ significantly between the non-allergic and allergic asthma patients. This finding is in keeping with the study demonstrating no differences in the serum BDNF levels between patients with allergic asthma and healthy individuals [23], or between children with allergic asthma and those without an atopic background [62], as well as with the report of no association between atopy and BDNF concentrations in the serum of patients with chronic cough [24]. However, our results did not mirror earlier studies showing that patients with allergic asthma have higher BDNF levels compared to control subjects [21,59]. Virchow et al. (1998) reported that BDNF is produced endobronchially following allergen provocation, suggesting a contribution to the pathogenesis of asthma [69]. In contrast to the study demonstrating lower amounts of BDNF in the eosinophil lysates of allergic asthma patients than control subjects [70], or the report on higher total BDNF in subjects with sputum eosinophilia [61], our study observed no differences in the plasma BDNF concentration between patients with and without eosinophilic asthma. As far as we are aware, our work is the first to compare the plasma BDNF levels between T2-low and T2-high asthma patients; therefore, our results showing no differences could not be evaluated against the literature data.

The *BDNF* Val66Met polymorphism has previously been associated with asthma in children of Chinese Han [7,30] and Slovak origin [15]; whereas a recent meta-analysis has suggested that the G allele of *BDNF* Val66Met polymorphism is potentially associated with asthma risk in Caucasians [71]. However, in our study, no significant differences in the frequencies of *BDNF* Val66Met genotypes, alleles or carriers were found between the asthma patients and control group. Our findings are consistent with reports of no association of *BDNF* Val66Met polymorphism with the presence of asthma in children [28], as well as with asthma-related phenotypes in families with asthma [29], suggesting that this *BDNF* gene variation does not contribute significantly to asthma susceptibility or severity. However, the same group of authors later reported that *BDNF* Val66Met polymorphism in a haplotype (TTGC) with three other *BDNF* polymorphisms may contribute to asthma susceptibility, but does not influence asthma severity [72]. These findings are in agreement with our study, which found no significant differences in the frequencies of *BDNF* Val66Met genotypes, alleles, and carriers between non-severe and severe asthma patients. In contrast, the study of Zeilinger et al. (2009) suggested that *BDNF* Val66Met polymorphism contributes to the severe forms of asthma [31]. 

Regarding *BDNF* Val66Met polymorphism, it has been shown that the Met variant alters the intracellular trafficking and packaging of pro-BDNF, resulting in reduced secretion of the mature BDNF [25,73]. However, various studies reported contradictory findings about the possible influence of functional *BDNF* Val66Met polymorphism on BDNF levels, demonstrating that carriers of the Met allele have decreased [74], increased [75], or unchanged [76] serum BDNF levels. In our study, healthy individuals carrying the Met allele had significantly lower plasma BDNF levels in comparison to the carriers of the Val/Val genotype, supporting the findings that *BDNF* Val66Met polymorphism influences BDNF concentration in plasma. On the other hand, this association was not found in asthma patients, as no significant differences in plasma BDNF levels were observed when the patients were subdivided according to *BDNF* Val66Met genotypes and carriers. Our results in asthma patients are in agreement with the data showing that *BDNF* Val66Met variants do not affect the BDNF serum levels in children with asthma [32]. It is possible that in patients with asthma, due to the complex pathophysiology of this disease, other factors predominantly influence BDNF plasma levels, masking the effect of the functional *BDNF* Val66Met polymorphism.

In the present study, there were no differences in the distribution of *BDNF* Val66Met genotypes, alleles or carriers between the non-allergic and allergic asthma patients. This is in line with the reported lack of difference in the genotype distribution for clinical atopy between asthmatic patients and healthy subjects [28], and with no association of *BDNF* Val66Met polymorphism with asthma-related atopy [29] or asthma and any atopic disease in the cross-sectional study population [31]. In contrast, some reports have suggested an important contribution of *BDNF* variations in the predisposition and progression of allergic diseases [77,78]. To the best of our knowledge, the distribution of *BDNF* Val66Met variants among different asthma phenotypes (T2-low vs. T2-high, eosinophilic vs. non-eosinophilic, and AERD vs. non-AERD) has not been investigated so far; therefore, our results showing no differences could not be discussed here in relation to the available literature. 

Our study observed no differences in the distribution of *NTRK2* rs1439050 genotypes, alleles, and carriers between healthy subjects and asthma patients, and between patients of different asthma severity or phenotypes. To the best of our knowledge, the association of *NTRK2* rs1439050 polymorphism with asthma, its severity and particular phenotypes has not been explored so far. However, some studies have examined other polymorphic variants within genes encoding neurotrophic receptors, including *TrkB* gene in asthma [20,32]. Some of the studied polymorphisms represent functional variants that may affect TrkB receptor expression. Higher TrkB expression has been found in asthma patients compared to healthy controls [20,60], and in bronchial eosinophils after segmental allergen provocation [79], but no differences in TrkB expression were observed between healthy subjects and patients with allergies in peripheral blood mononuclear cells [80]. Markedly increased mRNA levels of *TrkB* have also been detected in the inflamed lung tissue of a murine model of asthma [81]. In addition, cigarette smoke has been shown to be a potent inducer of TrkB expression and signaling in airway smooth muscle [58], thus contributing to airway hyperresponsiveness in allergic asthma [82]. In addition to the TrkB receptor levels, polymorphic variants of neurotrophic receptor genes might also affect the concentrations of unbound/active BDNF protein. However, similar to the findings of Szczepankiewicz et al. [32], in our study plasma BDNF levels were not influenced by *NTRK2* rs1439050 polymorphism in both healthy individuals and asthma patients. 

It is important to acknowledge several limitations of the present study. First, control subjects and asthma patients were not matched in their demographic parameters, such as age, gender, and smoking status. Another limitation of this study is that we did not gather any data about the physical activity and/or exercise regimen of the enrolled subjects. Specifically, increased peripheral lactate levels following high intensity exercise have been associated with increased peripheral BDNF concentrations [83]. Therefore, future studies should take into account the physical activity of participants and its potential influence on plasma BDNF levels. Moreover, the exclusion criteria of this study were quite restrictive and future studies should be conducted on much larger samples of asthma patients, taking into account the effects of comorbidities and medications, to reflect the real-world data. As the role of depression in asthma is an ongoing area of research [84,85,86], it would be important to also incorporate these patients in future studies. On the other hand, the present study included an adequate number of participants in order to have statistical power and to observe statistically significant associations. Another strength of the current study is that it enrolled an ethnically homogenous population, consisting of Caucasian Croatians, which is especially important for genetic studies. Additionally, this study took into consideration asthma severity and several asthma phenotypes (T2-high and T2-low, non-allergic and allergic, non-eosinophilic, and eosinophilic, as well as non-AERD and AERD), which have not been covered adequately in previous studies and should be studied more extensively in the future.

## 5. Conclusions

In conclusion, our findings indicate that asthma patients have higher plasma BDNF levels than healthy subjects. This may be due to a compensatory mechanism involving the activation of immune cells in asthma. According to our results, plasma BDNF levels cannot be used to distinguish severe from non-severe disease or between the different asthma phenotypes (allergic versus non-allergic, T2-high versus T2-low, or non-eosinophilic versus eosinophilic asthma). On the other hand, significantly and nominally higher plasma BDNF levels were observed in patients with aspirin-sensitive asthma and AERD, respectively. 

The genotype and allele frequencies of *BDNF* Val66Met and *NTRK2* rs1439050 polymorphisms did not differ between healthy individuals and asthma patients, nor between patients grouped according to severity or different asthma phenotypes. Plasma BDNF concentration was not influenced by *NTRK2* rs1439050 polymorphism in both healthy subjects and asthma patients. In contrast to the control group, plasma BDNF concentrations in the asthma patients were not affected by the *BDNF* Val66Met genotype, allele, or carrier distribution. Therefore, plasma BDNF levels in asthma patients are likely influenced by a complex interaction between environmental factors and genetic predisposition, and might be primarily related to the pathophysiology of the disease. Overall, our results suggest that BDNF may be a useful peripheral blood biomarker of asthma in general, and especially for asthma with aspirin sensitivity. However, additional studies, including larger numbers of subjects, are needed in order to assess the clinical impact of these findings. Moreover, further research is warranted to better understand the role of peripheral BDNF levels, as well as *BDNF* and *NTRK2* (*TrkB* gene) polymorphisms, in adult asthma.

## Figures and Tables

**Table 1:** Characteristics of asthma patients and healthy subjects.

Parameter	Asthma Patients (n = 120)	Healthy Controls (n = 120)	Statistical Analysis
**Age (years)** **Median (25%; 75%)**	58.00 (40.25; 67.00)	42.00 (33.25; 51.00)	*p* < 0.0001; U = 3912.00 Mann-Whitney test
**Males** **n (%)**	41 (34.17)	73 (60.83)	*p* < 0.0001 Fisher’s exact test
**Current smokers** **n (%)**	10 (8.33)	41 (34.17)	*p* = 0.0005 Fisher’s exact test
**BMI (kg/m^2^)** **Median (25%; 75%)**	26.65 (23.03; 29.80)	26.20 (23.33; 28.85)	*p* = 0.64; U = 6949.00 Mann-Whitney test
**BMI category: Normal weight** **(18.5–24.9 kg/m^2^), n (%)**	44 (36.67)	43 (35.83)	*p* = 0.48; χ^2^ = 4.52 χ^2^-test
**BMI category: Obesity** **(≥30 kg/m^2^), n (%)**	28 (23.33)	22 (18.33)	*p* = 0.43 Fisher’s exact test

BMI = Body mass index.

**Table 2:** Distribution of genotypes, alleles and carriers of *BDNF* Val66Met polymorphism in asthma patients and healthy subjects.

*BDNF* Val66Met Polymorphism		Asthma Patients n (%)	Healthy Controls n (%)	Statistical Analysis
**Genotypes**	**AA**	3 (2.50)	2 (1.67)	*p* = 0.54; χ^2^ = 1.23 χ^2^-test
	**AG**	43 (35.83)	36 (30.00)	
	**GG**	74 (61.67)	82 (68.33)	
**Alleles**	**A**	49 (20.42)	40 (16.67)	*p* = 0.35 Fisher’s exact test
	**G**	191 (79.58)	200 (83.33)	
**Carriers**	**A**	46 (38.33)	38 (31.67)	*p* = 0.34 Fisher’s exact test
	**GG**	74 (61.67)	82 (68.33)	
	**G**	117 (97.50)	118 (98.33)	*p* = 1.00 Fisher’s exact test
	**AA**	3 (2.50)	2 (1.67)	

**Table 3:** Plasma BDNF concentration in asthma patients and healthy subjects carrying different *BDNF* Val66Met genotypes and alleles.

*BDNF* Val66Met Polymorphism		Asthma Patients		Healthy Subjects	
		Plasma BDNF Concentration (pg/mL) Median (25%; 75%)	Statistical Analysis	Plasma BDNF Concentration (pg/mL), Median (25%; 75%)	Statistical Analysis
**Genotypes**	**AA**	0.45 (0.43; 1.24)	*p* = 0.52 Kruskal-Wallis test	0.56 (0.47; 0.65)	*p* = 0.03 Kruskal-Wallis test *
	**AG**	0.81 (0.52; 1.35)		0.51 (0.32; 0.73)	
	**GG**	0.94 (0.62; 0.94)		0.68 (0.49; 1.05)	
**Carriers**	**A**	0.80 (0.46; 1.34)	*p* = 0.39; U = 1544.00 Mann-Whitney test	0.51 (0.35; 0.70)	*p* = 0.008; U = 1089.00 Mann-Whitney test
	**GG**	0.94 (0.62; 1.20)		0.68 (0.49; 1.05)	
	**G**	0.91 (0.61; 1.22)	*p* = 0.37; U = 120.50 Mann-Whitney test	0.59 (0.45; 0.93)	*p* = 0.64; U = 94.50 Mann-Whitney test
	**AA**	0.45 (0.43; 1.24)		0.56 (0.47; 0.65)	

BDNF = Brain-derived neurotrophic factor; * AG vs. GG, *p* = 0.03 Dunn’s multiple comparisons test.

**Table 4:** Distribution of genotypes, alleles and carriers of *NTRK2* rs1439050 polymorphism in asthma patients and healthy subjects.

*NTRK2 rs1439050* Polymorphism		Asthma Patients n (%)	Healthy Controls n (%)	Statistical Analysis
**Genotypes**	**GG**	65 (54.17)	61 (50.83)	*p* = 0.86; χ^2^ = 0.29 χ^2^-test
	**GT**	49 (40.83)	52 (43.33)	
	**TT**	6 (5.00)	7 (5.83)	
**Alleles**	**G**	179 (74.58)	174 (72.50)	*p* = 0.68 Fisher’s exact test
	**T**	61 (25.42)	66 (27.50)	
**Carriers**	**G**	114 (95.00)	113 (94.17)	*p* = 1.00 Fisher’s exact test
	**TT**	6 (5.00)	7 (5.83)	
	**T**	55 (45.83)	59 (49.17)	*p* = 0.70 Fisher’s exact test
	**GG**	65 (54.17)	61 (50.83)	

**Table 5:** Plasma BDNF concentration in asthma patients and healthy subjects carrying different *NTRK2* rs1439050 genotypes and alleles.

*NTRK2 rs1439050* Polymorphism		Asthma Patients		Healthy Subjects	
		Plasma BDNF Concentration (pg/mL) Median (25%; 75%)	Statistical Analysis	Plasma BDNF Concentration (pg/mL), Median (25%; 75%)	Statistical Analysis
**Genotypes**	**GG**	0.81 (0.59; 1.15)	*p* = 0.13 Kruskal-Wallis test	0.59 (0.49; 0.94)	*p* = 0.42 Kruskal-Wallis test
	**GT**	0.97 (0.63; 1.37)		0.59 (0.39; 0.84)	
	**TT**	0.51 (0.41; 1.37)		0.65 (0.30; 1.48)	
**Carriers**	**G**	0.91 (0.61; 1.22)	*p* = 0.23; U = 241.5 Mann-Whitney test	0.59 (0.45; 0.90)	*p* = 0.52; U = 336.5 Mann-Whitney test
	**TT**	0.51 (0.41; 1.37)		0.65 (0.30; 1.48)	
	**T**	0.97 (0.58; 1.37)	*p* = 0.21; U = 1550 Mann-Whitney test	0.60 (0.39; 0.88)	*p* = 0.36; U = 1623 Mann-Whitney test
	**GG**	0.81 (0.59; 1.15)		0.59 (0.49; 0.94)	

**Table 6:** Distribution of genotypes, alleles and carriers of *BDNF* Val66Met polymorphism in non-severe and severe asthma patients.

*BDNF* Val66Met Polymorphism		Non-Severe Asthma Patients, n (%)	Severe Asthma Patients, n (%)	Statistical Analysis
**Genotypes**	**AA**	0 (0.00)	3 (4.92)	*p* = 0.13; χ^2^ = 4.04 χ^2^-test
	**AG**	19 (32.20)	24 (39.34)	
	**GG**	40 (67.80)	34 (55.74)	
**Alleles**	**A**	19 (16.10)	30 (24.59)	*p* = 0.11 Fisher’s exact test
	**G**	99 (73.90)	92 (75.41)	
**Carriers**	**A**	19 (32.20)	27 (44.26)	*p* = 0.19 Fisher’s exact test
	**GG**	40 (67.80)	34 (55.74)	
	**G**	59 (100.00)	58 (95.08)	*p* = 0.24 Fisher’s exact test
	**AA**	0 (0.00)	3 (4.92)	

**Table 7:** Distribution of genotypes, alleles and carriers of *NTRK2* rs1439050 polymorphism in non-severe and severe asthma patients.

*NTRK2 rs1439050* Polymorphism		Non-Severe Asthma Patients, n (%)	Severe Asthma Patients, n (%)	Statistical Analysis
**Genotypes**	**GG**	33 (55.93)	32 (52.46)	*p* = 0.56; χ^2^ = 1.16 χ^2^-test
	**GT**	22 (37.29)	27 (44.26)	
	**TT**	4 (6.78)	2 (3.28)	
**Alleles**	**G**	88 (74.58)	91 (74.59)	*p* = 1.00 Fisher’s exact test
	**T**	30 (25.42)	31 (25.41)	
**Carriers**	**G**	55 (93.22)	59 (96.72)	*p* = 0.43 Fisher’s exact test
	**TT**	4 (6.78)	2 (3.28)	
	**T**	26 (44.07)	29 (47.54)	*p* = 0.72 Fisher’s exact test
	**GG**	33 (55.93)	32 (52.46)	

**Table 8:** The association of plasma BDNF concentration with clinical characteristics in asthma patients.

Clinical Characteristics	Median (25%; 75%)	Plasma BDNF Concentration (pg/mL)
**Total Serum IgE (IU/mL)**	191.00 (43.00; 398.00)	*p* = 0.50; Spearman correlation r = −0.06
**Blood eosinophils (×10^9^/L)**	0.215 (0.10; 0.40)	*p* = 0.69; Spearman correlation r = 0.04
**Blood neutrophils (×10^9^/L)**	4.560 (3.62; 6.30)	*p* = 0.92; Spearman correlation r = −0.01
**FeNO (ppb)**	34.00 (14.28; 67.75)	*p* = 0.98; Spearman correlation r = −0.002
**FEV_1_ (% of predicted value)**	74.90 (54.28; 90.08)	*p* = 0.41; Spearman correlation r = 0.07
**FVC (% of predicted value)**	88.50 (75.08; 101.80)	*p* = 0.65; Spearman correlation r = −0.04
**PEF (% of predicted value)**	77.05 (57.25; 95.52)	*p* = 0.58; Spearman correlation r = −0.05
**DLCO (%)**	83.30 (73.68; 95.35)	*p* = 0.055: Spearman correlation r = −0.17
**Duration of disease (years)**	13.50 (8.00; 29.00)	*p* = 0.023; Spearman correlation r = 0.21
**Comorbidities (n)**	2.00 (1.25; 4.00)	*p* = 0.35; Spearman correlation r = 0.08
**Clinical Characteristics**	**n (%)**	**Plasma BDNF Concentration (pg/mL)**
**Penicillin allergy**	25 (20.83)	*p* = 0.18; U = 977.50; Mann-Whitney test
**Nutritive allergy**	12 (10.00)	*p* = 0.10; U = 458.50; Mann-Whitney test
**Animal dander/feather allergy**	18 (15.00)	*p* = 0.31; U = 779.00; Mann-Whitney test
**Dust allergy**	51 (42.50)	*p* = 0.06; U = 1408.00; Mann-Whitney test
**Pollen allergy**	53 (44.17)	*p* = 0.72; U = 1709.00; Mann-Whitney test
**Fungal/mold allergy**	9 (7.50)	*p* = 0.28; U = 390.50; Mann-Whitney test
**Early onset asthma (age < 12 years)**	21 (17.50)	*p* = 0.12; U = 817.00; Mann-Whitney test
**History of pneumonia**	21 (17.50)	*p* = 0.026; U = 718.00; Mann-Whitney test
**Emergency intervention (ever)**	89 (74.17)	*p* = 0.53; U = 1274.00; Mann-Whitney test
**Hospitalization for asthma (ever)**	58 (48.33)	*p* = 0.70; U = 1724.00; Mann-Whitney test
**Nasal polyps**	27 (22.50)	*p* = 0.025; U = 891.00; Mann-Whitney test
**Aspirin sensitivity**	11 (9.17)	*p* = 0.009; U = 314.00; Mann-Whitney test
**Allergen specific immunotherapy**	15 (12.50)	*p* = 0.99; U = 787.00; Mann-Whitney test
**Oral corticosteroid therapy**	30 (25.00)	*p* = 0.58; U = 1258.00; Mann-Whitney test
**Biological therapy**	20 (16.67)	*p* = 0.80; U = 962.50; Mann-Whitney test

DLCO = diffusing capacity of lung for carbon monoxide; FeNO = fractional exhaled nitric oxide; FEV_1_ = forced expiratory volume in one second; FVC = forced vital capacity; IgE = immunoglobulin E; PEF = peak expiratory flow; ppb = parts per billion.

**Table 9:** Plasma BDNF concentration in patients with different asthma phenotypes.

Asthma Phenotypes	Plasma BDNF Concentration (pg/mL) Median (25%, 75%)	Statistical Analysis
**T2-high (n = 94)**	0.91 (0.60; 1.30)	*p* = 0.43; U = 1097.00 Mann-Whitney test
**T2-low (n = 26)**	0.82 (0.42; 1.14)	
**Non-allergic (n = 42)**	0.98 (0.60; 1.29)	*p* = 0.41; U = 1487.00 Mann-Whitney test
**Allergic (n = 78)**	0.82 (0.58; 1.20)	
**Non-eosinophilic (n = 73)**	0.92 (0.54; 1.20)	*p* = 0.54; U = 1602.00 Mann-Whitney test
**Eosinophilic (n = 47)**	0.88 (0.61; 1.34)	
**Non-AERD (n = 111)**	0.85 (0.58; 1.18)	*p* = 0.017; U = 262.00 Mann-Whitney test
**AERD (n = 9)**	1.22 (0.92; 2.05)	

AERD = aspirin-exacerbated respiratory disease.
